# Comorbidity and quality of life in obesity–a comparative study with the general population in Gothenburg, Sweden

**DOI:** 10.1371/journal.pone.0273553

**Published:** 2022-10-04

**Authors:** Ala Mejaddam, Emily Krantz, Gudrún Höskuldsdóttir, Lars Fändriks, Karin Mossberg, Björn Eliasson, Penelope Trimpou, Kerstin Landin-Wilhelmsen

**Affiliations:** 1 Department of Internal Medicine, Sahlgrenska University Hospital/Östra, Gothenburg, Sweden; 2 Department of Internal Medicine and Clinical Nutrition, Institute of Medicine, Gothenburg, Sweden; 3 Department of Respiratory Medicine, Sahlgrenska University Hospital, Gothenburg, Sahlgrenska Academy, University of Gothenburg, Gothenburg, Sweden; 4 Section for Endocrinology and Diabetology, Sahlgrenska University Hospital, Gothenburg, Sweden; 5 Department of Surgery, Institution for Clinical Sciences, Sahlgrenska Academy, University of Gothenburg, Gothenburg, Sweden; 6 Department of Public Health and Community Medicine Primary Health Care, Institute of Medicine, Sahlgrenska Academy, University of Gothenburg, Gothenburg, Sweden; Universiti Malaya, MALAYSIA

## Abstract

**Context:**

Obesity is considered to have a detrimental impact on health-related quality of life (HRQoL).

**Objective:**

To compare HRQoL in a well-defined group of people with obesity with a population-based control group from the general public.

**Design:**

Observational cross-sectional cohort study with a reference population.

**Setting:**

The Regional Obesity Center at the Department of Medicine at Sahlgrenska University Hospital, Gothenburg, Sweden.

**Participants:**

People with obesity (n = 1122) eligible for surgical and non-surgical obesity treatment in routine care were included consecutively between 2015 and 2017 into the BASUN study. Men and women from the WHO-MONICA-GOT project were used as a reference population (n = 414).

**Main outcome measures:**

HRQoL was measured with the RAND-36/Short Form-36 questionnaire (SF-36) and a Visual Analogue Scale (VAS) for self-related health (SRH). Prescription drugs for hypertension, diabetes mellitus, depression, and anxiety were taken as a proxy for these conditions.

**Results:**

People with obesity rated their overall HRQoL lower than the reference population according to the SRH-VAS. Lower scores were reported on physical and social functioning, vitality, general and mental health after adjustment for age and use of prescription drugs (considered a proxy for burden of disease, or comorbidities) using the RAND-36/SF-36 questionnaire. Use of some psychopharmacological agents was more common in patients with obesity.

**Conclusion:**

People with obesity seeking help with weight reduction are more likely to have lower physical and mental self-reported HRQoL than the general population.

## Introduction

Over the past four decades, obesity has become one of the world’s most serious public health challenges, with a global burden that has doubled during this time [1,2]. In the European Union, the latest estimates show that 30–70% of the adult population are overweight and 10–30% are obese [1]. This has led to calls for immediate actions to mitigate the obesity epidemic since obesity is a known risk factor for developing cardiovascular disease and type 2 diabetes mellitus, and even for an increased risk of premature death [2–4]. According to data from the Global Burden of Disease study in 2015, it was estimated that approximately 4 million deaths globally were related to a high body mass index (BMI) [1,2].

Obesity is associated with depression, anxiety disorders and impaired health related quality of life (HRQoL) [5,6]. Previous research on HRQoL suggest an association between obesity and impairments in most components of HRQoL [6]. However, previously published literature has been inconsistent on the relationship between higher BMI and lower HRQoL with some studies showing strong links with HRQoL, especially physical aspects, and other studies revealing weak, or no correlation at all with mainly mental HRQoL [7–10]. Differences in HRQoL have also been observed between treatment seeking groups and those with obesity not seeking professional help for weight reduction, indicating lower rates on overall quality of life in the former group, especially among those with morbid obesity (BMI ≥40 kg/m^2^) [11,12]. It has also been suggested that people with obesity do not have a lower well-being compared to normal weight individuals [13]. However, studies comparing HRQoL in people with obesity to the general population are rare.

More studies on individuals with obesity and their characteristics are essential for better understanding and management of obesity. This study is part of an ongoing prospective long-term project on the effects of weight reduction treatment to more closely look at a well-defined cohort of people with obesity before and after they received treatment. The primary aim of this study was to compare HRQoL in a cohort of people with obesity with a randomly selected reference group of men and women from the general population in the same region. The hypothesis was that people with obesity have a lower physical and mental well-being than that of the general population. Another specific aim was to compare comorbidities between the same groups, using prescribed drugs as a proxy for different comorbidities. The hypothesis was that people with obesity were prescribed diabetic and antihypertensive drugs, antidepressants, analgesics, anxiolytics and/or sedatives to a higher degree than in the general population.

## Materials and methods

### Study setting

This is an observational cross-sectional cohort study with a population-based sample as reference. It is based on two studies; i) an ongoing 10-year prospective cohort study including individuals with obesity called the BAriatric surgery SUbstitution and Nutrition study (BASUN) [14] and ii) a population-based cohort study conducted by the World Health Organization (WHO) called the MONItoring of trends and determinants in CArdiovascular disease (WHO MONICA) [15,16]. All subjects completed their medical examinations and questionnaires at the Sahlgrenska University Hospital, Gothenburg, Sweden.

### Subjects

#### BASUN cohort

Individuals with obesity that were referred to the Regional Obesity Centre (ROC), at the Sahlgrenska University Hospital in Gothenburg were included consecutively between 2015 and 2017 to the BASUN study. The ROC coordinates and assesses all referrals for obesity treatment in Region Västra Götaland, a county in western Sweden [14]. All patients underwent a thorough evaluation by a multidisciplinary board at the ROC and were approved for treatment prior to receiving enquiries regarding participation in the study.

Inclusion criteria for BASUN were eligibility for bariatric treatment according to international guidelines for bariatric surgery: a BMI >35 kg/m^2^ and the presence of obesity-related medical conditions, such as diabetes mellitus type 2, or patients with a BMI ≥40 kg/m^2^, even without such comorbidities [17]. Exclusion criteria were drug or alcohol abuse, unstable psychiatric disorders, age under 18 years, cancer during the last 5 years or presence of diseases threatening life in the short term [14,18]. Patients who were eligible for treatment but lacked adequate knowledge of the Swedish language were also excluded.

2,260 patients were considered eligible for participation but 1140 declined to participate (no further analyses were performed on these patients). In total, 1122 met the inclusion criteria, consented to participation and underwent examination at baseline including assessments with blood tests, anthropometric measurements, and questionnaires [14].

#### Reference population

In 1995, the third population screening of risk factors was undertaken by the WHO, as part of the WHO MONICA project. It was a collaboration of centers in 38 countries around the world, including Gothenburg, Sweden [15,16]. In Gothenburg, Sweden, 2563 individuals were recruited from the city census in 1995, which is kept up to date within a maximum of 14 days, and of these, 1618 individuals participated [16]. From the 1995 cohort, a subset of individuals was randomly selected and invited for a re-examination in 2008 (every 4^th^ subject, and all of the women between the ages 45–64 years; in total 662 subjects). 414 subjects participated in the re-evaluation and assessment of HRQoL [15]. The non-attendees were those who were deceased, did not reply or could not be traced, declined consent or failed to appear in clinic [15]. In this study, the cohort of n = 414 from the 2008 follow-up of the WHO MONICA project was used as reference population for the BASUN cohort.

#### Social variables and anthropometry

Data on age, sex, education level, marital status, smoking habits and physical activity was recorded [14,15]. The degree of physical activity was measured during leisure time and was based on a 4 point ordinal scale developed by Saltin and Grimby, in which the lowest grade of 1 indicates complete inactivity and the highest grade of 4 indicates strenuous activity several times a week [19]. Data regarding height, body weight and BMI was collected similarly in both groups. Height was measured without shoes to the nearest 1 cm and body weight was measured to the nearest 0.1 kg. BMI was calculated as body weight divided by height squared [14,15]. According to current definitions, BMI >25kg/m^2^ is considered overweight, BMI >30 kg/m^2^ is considered obese, and BMI ≥40 kg/m^2^ morbid obesity [2].

### Pharmacological treatment

Medical history was taken and recorded. Ongoing pharmacological treatments were coded according to the Anatomical Therapeutic Chemical (ATC) classification system for all participants. Self-reported use of prescription drugs for hypertension, diabetes mellitus, depression, anxiety, sleep disorders were taken as a proxy for each of these conditions.

### Assessment of quality of life

Two generic instruments that measure broad aspects of health-related quality of life (HRQoL) were used for both the study group and reference population. One of these instruments was the Visual Analogue Scale (VAS) for self-rated health (SRH) from the EuroQol Research Foundation in the 1990 edition questionnaire^TM^, EuroQol five-dimensional instrument (EQ-5D). The SRH-VAS is a single question requiring subjects to rate their current general health on a vertical linear scale, ranging from 0 to 100. The best imaginable level of health generating 100 points and the worst imaginable level of health at 0 [20]. SRH is an important indicator of general well-being and this scale has been shown to correlate well with generic multi-item questionnaires for HRQoL [21].

HRQoL was assessed using the RAND-36 in the BASUN study group and the Medical Outcome Study Short Form-36 (SF-36) questionnaire in the WHO-MONICA study group.

The SF-36/RAND-36 questionnaire is a multi-dimensional health survey comprised of eight domains with the purpose of representing the full range of health states. The eight scales include physical functioning, role limitations due to physical problems, bodily pain, general health, vitality, social functioning, mental health and role limitations due to emotional problems. The scales range from 0 to 100, a lower score indicating a greater impairment. The items, *i*.*e*. the questions posed to the participants, in SF-36 and RAND-36 are identical, but there is a difference in the algorithms used to measure the summary scores based on different assumptions on the relationship between physical and mental well-being [22,23]. Studies comparing these two versions in the same population show that they yield very similar results for all the eight domains but some differences can be seen between the summary scores [22–24]. Therefore, only the subscales, and not the summary scores, were compared and reported between the two groups. The summary scores were, however, included in the within obese group analyses.

Both SRH-VAS and especially, SF-36, are widely utilized generic instruments in studies assessing HRQoL in general and obese populations [6,25–27]. Studies have shown that both instruments have a strong discriminative ability between groups with known differences in health and can also reflect changes in quality of life [21,28,29].

### Ethical considerations

The BASUN study was approved 2014-09-24 (Dn. no 673–14) and The WHO MONICA Study approved 2006-05-22 (Dn. no 088–06 with addendum Ad 088–06, T 282–11 approved 2011-03-21) by the Ethical Review Board in Gothenburg. All participants gave their informed consent. Human rights were respected in accordance with the Helsinki Declaration.

### Statistical methods

Conventional methods were used to calculate means with standard deviation (SD) and range for continuous variables. Categorical values are reported as numbers with their percentages. The percentage of subjects with at least one prescription of each drug was calculated. Standardized mean difference (SMD) was then used to describe the differences between the patients with obesity (BASUN) and the reference population (WHO-MONICA study), as well as the difference between different BMI categories within the BASUN study. SMD is a measurement for effect size and is calculated by dividing the difference between sample means by the pooled SD. SMD values < 0.1 are considered non-significant [30]. For HRQoL, a multiple linear regression model was built to evaluate the association between HRQoL and different weight categories. First, we examined HRQoL between the patients with obesity in BASUN and the reference population. The same model was used to examine HRQoL in patients with BMI greater and lesser than 40 kg/m^2^ within BASUN. The model was adjusted for age and number of medications as both variables were deemed important determinants for the outcome. The results were presented as beta coefficients with 95% confidence intervals. All test were two-sided and a p-value <0.05 was considered statistically significant. No imputations were performed for missing data. A supplementary table is added for the number of subjects with missing data for each variable. All statistical analyses were calculated using R (R Foundation for Statistical Computing, version 4.0.3) and Microsoft Excel.

### Main results

A clinical description of patients with obesity and the reference population is presented in Table 1. There were no differences between the groups regarding distribution of gender. People in the reference population were older and had lower BMI (SMD>0.1). Only 6% in the reference population had a BMI of more than 35 kg/m^2^.

**Table 1:** Anthropometric and social background data, degree of physical activity, number of medications and data on those who used at least one prescription drug in people with obesity (BASUN) and the reference population from the WHO-MONICA study, Gothenburg, Sweden.

	Patients with obesity (n = 1122)	WHO MONICA, Controls (n = 414)	*p-value*	SMD
Sex, Female, n (%)	828 (73.8)	318 (76.8)	0.30	0.10
Age, years (range; SD)	43.9 (18–78; 12.9)	62.8 (39–78; 9.4)	<0.001	1.67
BMI, kg/m^2^, mean (range; SD)	42.0 (31.6–90.4; 5.0)	26.8 (17.2–54.8, 4.7)	<0.001	3.15
Physical exercise, n (%)			<0.001	0.82
Sedentary	431 (44.3)	55 (13.5)		
Moderate	470 (48.4)	246 (60.4)		
Regular/heavy	71 (7.0)	106 (26.0)		
Smokers, n (%)	66 (7.6)	46 (11.3)	0.04	0.12
Married/cohabiting, n (%)	613 (64.7)	348 (86.4)	<0.001	0.52
Completed secondary school, n (%)	814 (87.3)	197 (51.8)	<0.001	0.84
Number of medications, mean/person, n (range; SD)	3 (0–20; 3.0)	2 (0–10; 2.3)	0.005	0.17
Antidepressant agents, n (%)	216 (19.3)	43 (10.4)	<0.001	0.25
Anxiolytic and sedative agents, n (%)	22 (2.0)	49 (11.8)	<0.001	0.40
Analgetic agents, n (%)	178 (15.9)	34 (8.2)	<0.001	0.24
Lipid lowering agents, n (%)	127 (11.3)	62 (15.0)	0.07	0.11
Antihypertensive agents, n (%)	315 (28.1)	118 (28.5)	0.92	0.01
Glucose lowering agents, n (%)	139 (12.4)	10 (2.4)	<0.001	0.39

Data is given in means (range/SD) or n (%). BMI, Body mass index. SMD, standardized mean difference, values >0.1 or *p-value* <0.05 were considered statistically significant.

The number of prescribed medications was higher among the patients with obesity compared to the reference population, but the degree of physical activity was lower during leisure time (SMD>0.1). The rates of smoking, cohabitation, and education are also shown in Table 1. At the initial WHO MONICA screening in 1995, the reference population had an average age of 46 years (range 25–65), similar to the patients with obesity here, and an average BMI of 25 kg/m^2^. They also had a lower number of prescribed medications and were more physically active (data not shown, data available upon request) [16].

In the BASUN cohort, a higher number of the patients were prescribed glucose lowering medication, antidepressants and pain medications (SMD>0.1), while the people in the reference population were more likely to be treated with anxiolytics or sedatives (SMD>0.1). There was no difference in prescription of antihypertensive or lipid lowering drugs (Table 1).

### HRQoL in patients with obesity vs. reference population

**Fig 1:**
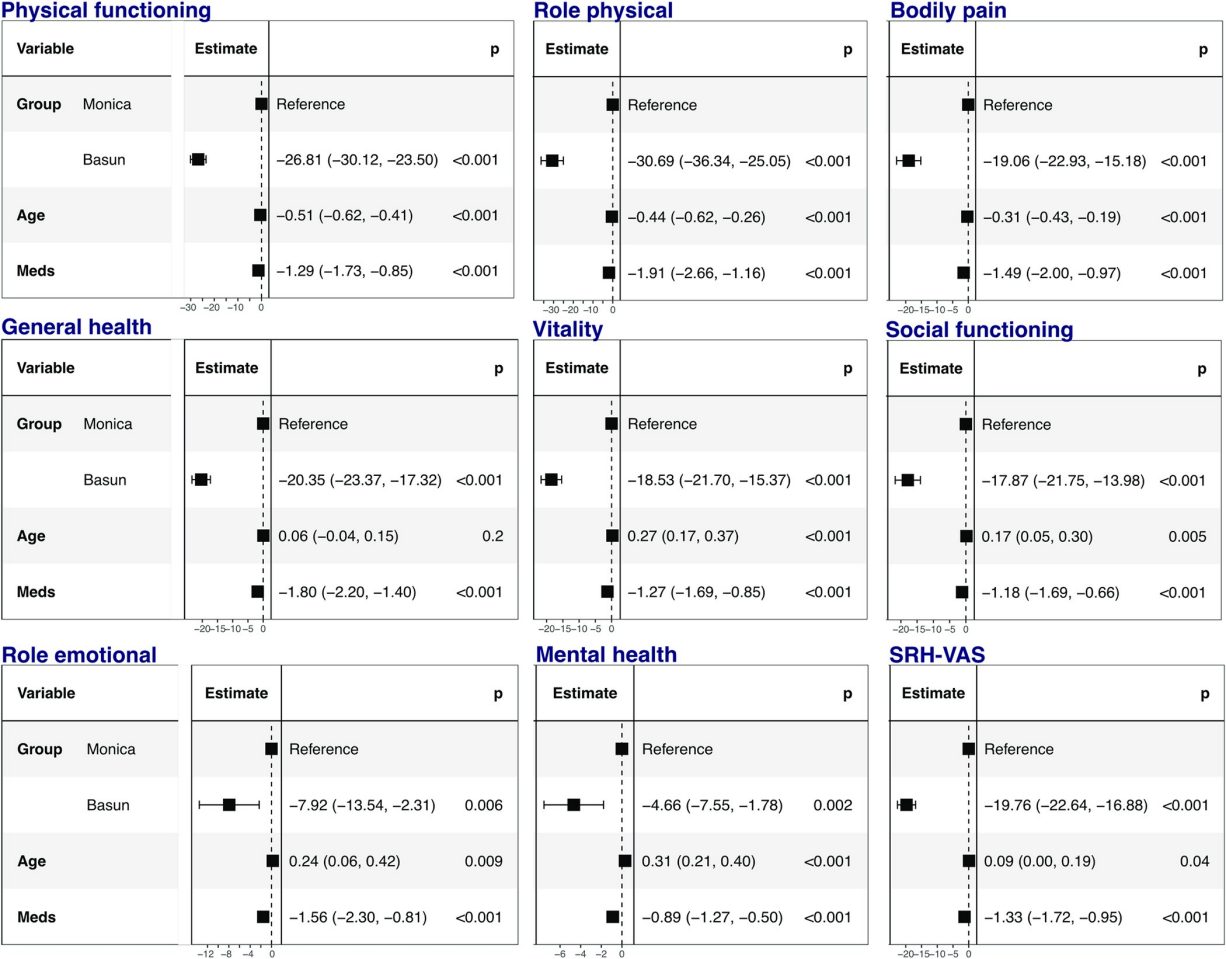
Results from a multiple linear regression for evaluation of association between health-related quality of life indicated by the Rand-36 and SRH-VAS instruments and weight category (BASUN population with obesity and reference MONICA population). The model is adjusted for age and number of medication (Meds) and results presented as beta coefficients with 95% confidence intervals.

87% of the patients with obesity and 99% of the subjects in the reference group completed the HRQoL questionnaires. The patients with obesity had a lower general well-being according to the SRH-VAS scale category than the reference population. They also reported lower scores on physical and social functioning, as well as lower vitality, general and mental health (Table 2 and Fig 1), (p<0.001, SMD>0.1). These results were similar also after adjustments for age and number of medications in the regression analyses, respectively (Fig 1).

**Table 2:** Health-related quality of life (HRQoL) measured with RAND-36/Short Form-36 (SF-36) and SRH-VAS in people with obesity (BASUN) and a reference population from the WHO-MONICA study, Gothenburg, Sweden. Mean values are given for all subscales.

HRQoL	People with obesity (n = 1122)	WHO MONICA, Controls (n = 414)	*p-value*	SMD
Age, years (SD)	43.9 (12.9)	62.8 (9.4)	<0.001	1.67
Number of medications, mean/person, n (SD)	3 (3.0)	2 (2.3)	0.005	0.17
RAND-36/SF-36, mean (SD)				
*Physical functioning*	59.8 (24.6)	78.0 (23.7)	<0.001	0.75
*Role physical*	50.7 (41.0)	74.5 (37.2)	<0.001	0.61
*Bodily pain*	55.3 (28.3)	69.5 (25.7)	<0.001	0.53
*General health*	46.8 (20.9)	69.3 (23.4)	<0.001	1.01
*Vitality*	41.9 (21.8)	66.1 (23.6)	<0.001	1.07
*Social functioning*	63.8 (28.7)	85.6 (22.7)	<0.001	0.84
*Role emotional*	65.5 (40.6)	78.8 (35.3)	<0.001	0.35
*Mental health*	66.8 (20.5)	77.6 (19.9)	<0.001	0.54
SRH-VAS, mean (SD)	53.4 (20.1)	75.7 (20.4)	<0.001	1.10

SRH-VAS, self-related health according to the visual analogue scale in the EQ-5D questionnaire. SMD, standardized mean difference, values >0.1 or *p-value* <0.05 were considered statistically significant.

### HRQoL in the patients with obesity

**Fig 2:**
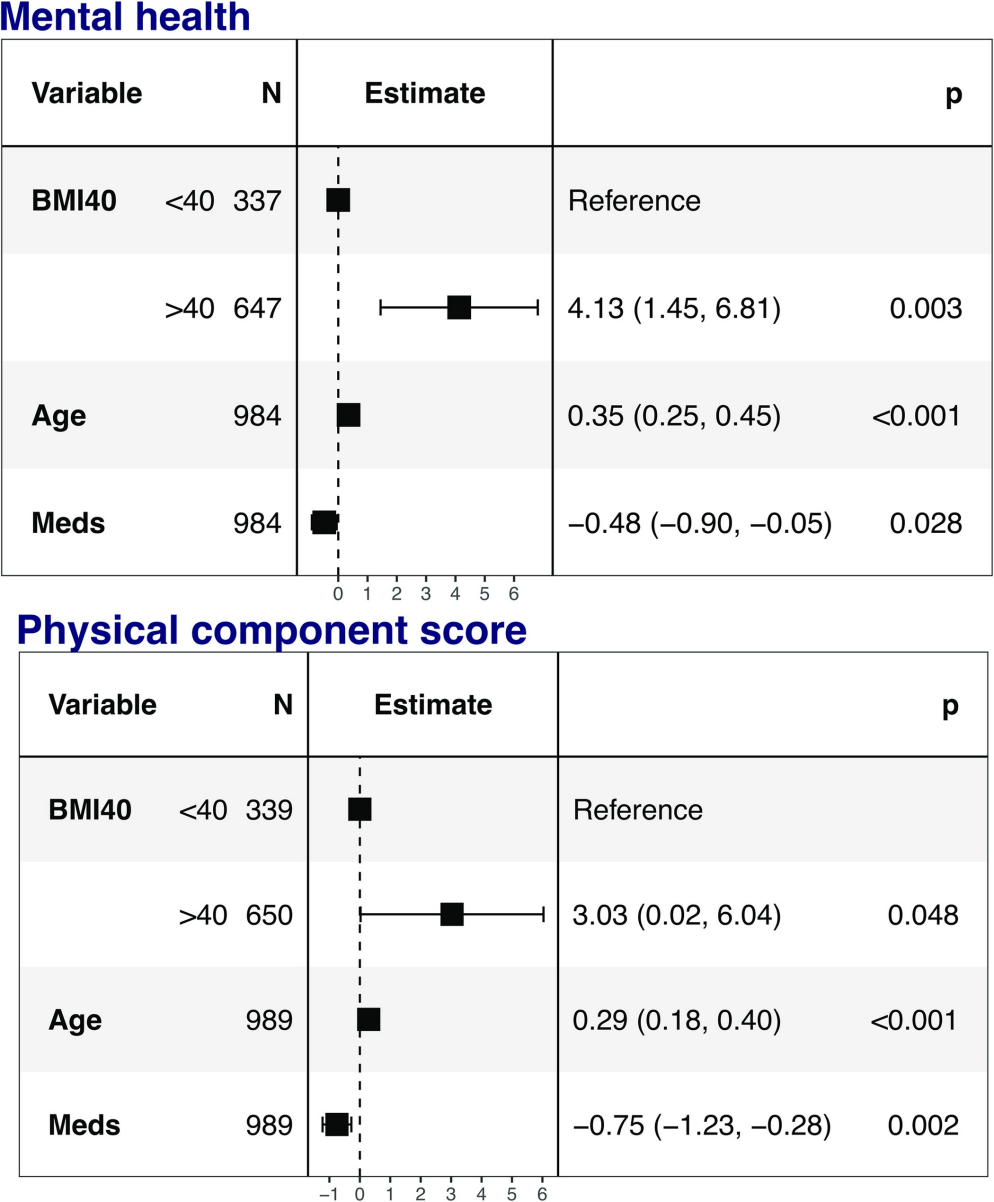
Results of a multiple linear regression for evaluation of association between health-related quality of life indicated by the Rand-36 instrument and BMI category (BASUN population with obesity) for the Mental health and Physical Component Score (PCS) domains. The model is adjusted for age and number of medication (Meds) and results presented as beta coefficients with 95% confidence intervals.

The study group with obesity was divided into two BMI categories (BMI <40 kg/m^2^ and BMI ≥40 kg/m^2^) for further analysis on differences in HRQoL between the subgroups. There were no significant differences between the two groups in most of the HRQoL scales used (Table 3). The difference in PCS between the two BMI categories did not remain after adjustment for age and number of medications (Fig 2). The group with a higher BMI did not have an increased number of prescribed medications taken daily (p<0.001, Table 3) due to the inclusion criteria. Only 2% (n = 25) in BASUN had a BMI less than 35 kg/m^2^ and they were included in the study because of higher burden of disease.

**Table 3:** Health-related quality of life (HRQoL) between two different BMI categories in the group with obesity (BASUN). Mean values are given for all RAND-36 scales and SRH-VAS, unless otherwise specified.

	BMI <40 kg/m^2^ (n = 384)	BMI ≥40 kg/m^2^ (n = 737)	*p-value*	SMD
Age, years (SD)	46.0 (13.1)	42.7 (12.6)	<0.001	0.26
Number of medications, n (SD)	3.1 (3.3)	2.2 (2.8)	<0.001	0.27
RAND-36, mean (SD)				
Physical functioning	61.6 (25.3)	58.8 (24.2)	0.09	0.11
Role physical	49.6 (41.0)	51.3 (41.1)	0.52	0.04
Bodily pain	56.2 (28.4)	54.8 (28.3)	0.46	0.05
General health	46.4 (21.3)	47.1 (20.6)	0.63	0.03
Vitality	40.6 (21.0)	42.5 (22.1)	0.19	0.09
Social functioning	62.2 (29.8)	64.7 (28.2)	0.20	0.09
Role emotional	63.8 (41.5)	66.4 (40.2)	0.35	0.06
Mental health	64.7 (22.1)	67.9 (19.6)	0.02	0.15
PCS	54.8 (18.1)	60.3 (22.4)	<0.001	0.11
MCS	53.5 (22.7)	53.1 (22.9)	0.78	0.02
SRH-VAS, mean (SD)	53.9 (19.7)	53.1 (20.4)	0.58	0.04

SRH-VAS, self-related health according to the visual analogue scale in the EQ-5D questionnaire. PCS, physical component score. MCS, mental component score. SMD, standardized mean difference, values >0.1 or *p-value* <0.05 were considered statistically significant.

## Discussion

The main finding of this study was that patients with obesity had a lower overall well-being (SRH) and lower both physical and mental HRQoL before weight lowering treatment was initiated compared to a representative cohort of people from the general population. Previous studies examining the impact of obesity on HRQoL have shown that HRQoL is affected within this group. However, the present study is one of only few that compare HRQoL in treatment seeking individuals with obesity with men and women from the general population [7,8,25,31–33]. A controlled study like this one strengthens previous data suggesting that this group of patients who seek treatment for obesity is more likely to have impaired HRQoL, and not only when compared to groups of people with similar body weight [12].

The majority of the subjects in this study suffered from morbid obesity (66% with BMI ≥40kg/m^2^). According to a meta-analysis by Ul-Haq et al, impairments on mental health are more likely associated with morbid obesity [8], which is in line with our findings of significantly impaired mental HRQoL as assessed by SF-36/RAND-36 in three of the four mental domains compared to the general population. When HRQoL in those with morbid obesity was compared to those with BMI <40 kg/m^2^ in the BASUN cohort, there were no significant differences found in most of the HRQoL domains. This suggests that other factors than just BMI may account for the lower HRQoL in patients with morbid obesity.

People with, and without obesity alike, who have a higher burden of disease have been shown to have a lower physical and emotional HRQoL than those without comorbidities [7,13,33,34]. In this study the patients with obesity used significantly more prescription drugs than the subjects in the reference group, indicating a higher burden of disease despite the, on average, 19-year age difference between the groups, but this only partly explains the difference in HRQoL between the groups. Gender difference is considered a determinant for HRQoL as well, with women reporting higher rates of impaired quality of life, especially mental health [9,35]. This could also be an explanation of the results in the present study showing both poorer mental and physical HRQoL, since women constituted nearly 80 percent of the study group. However, the sex distribution was similar in both patients and controls. It has previously been suggested that obese individuals seeking surgical treatment exhibit a more severely reduced overall HRQoL compared to obese individuals not seeking treatment [12,36]. For example, in two baseline studies for bariatric surgery candidates by Mitchell *et al*. and Dreber *et al*., subjects seeking treatment are characterized as having higher rates of psychiatric disorders and poorer mental health [5,37]. The present findings support that conclusion. However, neither Mitchell *et al*. nor Dreber *et al*. compared their results to a general population sample. It has also been implied that the low HRQoL in those seeking healthcare could provide motivation to undergo invasive treatment [6]. Identifying factors most likely to affect HRQoL in patients with obesity seeking weight loss treatment, plays a large role in the effectiveness of treatment and the path to good recovery.

Psychiatric illness is strongly associated with lower emotional well-being in people with obesity [5,25,36,37]. The use of antidepressants was more common among the patients with obesity in this study compared to the reference population. This indicates a higher prevalence of psychiatric morbidity which may also be an explanation for the difference in HRQoL found between the groups. Because of the cross-sectional design of this study, it is impossible to draw any causative conclusions on psychiatric illness, obesity and HRQoL. There also seems to be a bi-directional relationship between obesity and psychiatric diseases [38]. An implication of the present findings, in addition to the previous body of knowledge, is that psychiatric comorbidities might limit the positive effects of potential weight reduction on overall HRQoL in this population if not appropriately addressed.

Use of analgesics was also more common in the group with obesity than in the reference population. Despite this, they did not report more bodily pain in the SF-36/Rand-36 questionnaire. One explanation could be that the patients with obesity did not experience increased physical pain because they were being successfully treated with medication. On the other hand, they reported a lower degree of physical activity compared to the reference population and lower scores in Physical functioning scale which might indicate that they were less likely to engage in physical activities. This could, besides more use of analgesics, possibly explain why they perceived less pain. Physical inactivity has also been shown to negatively impact HRQoL independent of weight status [39].

### Strengths and limitations

To the best of our knowledge, few comparative baseline studies have been published on cohorts of patients with obesity before intervention [14]. An important strength of the study was the use of a reference population that was randomly selected from the general population from the same region. Another strength was the large sample size of the two cohorts. All the participants in the group with obesity were recruited through medical referral which may affect the generalizability of the results on the entire population with obesity. However, patient and clinician involvement in the recruitment process is representative of real life and those who seek help and is therefore a good representation of obese patients seeking treatment. Furthermore, two different generic HRQoL instruments were used with similar outcomes, which strengthens the validity of the results [21,25]. A general limitation was the cross-sectional study that does not allow us to draw conclusions on causality. The reference group was neither age-matched nor concurrent with the patient study group and caution is therefore warranted when interpreting the results of the study. Since societal behaviors and systems are likely to be different at different points in history, secular trends, and the age disparity in addition to using a historical comparison group, increases the risk for influence by confounding factors. Another limitation was the inclusion of people with obesity with metabolic comorbidities if they had a BMI between 30 and 40 kg/m^2^ which may affect the results even if adjustments for both age and disease burden were performed. There was no data collected on those that declined participation in the BASUN project. Finally, the sole use of generic instruments for evaluation of HRQoL may not be sensitive to the specific health concerns perceived by obese individuals but do allow for comparisons with a reference population.

## Conclusion

Patients with obesity seeking weight loss treatment are more likely to suffer from mental illness and exhibit poorer HRQoL than the general population. The interaction between obesity, HRQoL and psychiatric illness, and its significance for the results in the treatment of obesity are not yet clear. It will be interesting to study whether treatment intervention for obesity and normalized BMI affect HRQoL and prevalence of mental illness in patients with obesity compared to the general population afterwards, and whether prior HRQoL and presence of mental illness affect the outcome of the treatment.

## Supporting information

S1 Table Number of subjects with missing data for each studied variable. (DOCX)

S1 File Raw data for BASUN. (XLSX)

S2 File Raw data for WHO-MONICA. (XLSX)
