# Supplementary material for: Comorbidity and quality of life in obesity–a comparative study with the general population in Gothenburg, Sweden
Source: PLoS One. 2022 Oct 4;17(10):e0273553. doi: 10.1371/journal.pone.0273553 (PMC9531784; doi:10.1371/journal.pone.0273553)
Supplement: S1 Table — (DOCX) [file pone.0273553.s001.docx]

| **Supplementary table.** Number (n) of subjects with missing data for each studied variable; anthropometric and social background data, physical activity, prescription drugs and health related quality of life. | | |
| --- | --- | --- |
|  | **Patients with obesity**  **(n = 1122)** | **WHO MONICA,**  **Controls**  **(n = 414)** |
| **Sex** | 3 | 0 |
| **Age, years** | 0 | 0 |
| **Body mass index, kg/m²** | 0 | 1 |
| **Physical exercise, Saltin Grimby** | 149 | 7 |
| **Smokers** | 256 | 6 |
| **Married/cohabiting** | 173 | 11 |
| **Completed secondary school** | 189 | 34 |
| **Number of medications per person** | 0 | 0 |
| **Antidepressant agents** | 0 | 0 |
| **Anxiolytic and sedative agents** | 0 | 0 |
| **Analgetic agents** | 0 | 0 |
| **Lipid lowering agents** | 0 | 0 |
| **Antihypertensive agents** | 0 | 0 |
| **Glucose lowering agents** | 0 | 0 |
| **RAND-36/SF-36** |  |  |
| *Physical functioning* | 129 | 3 |
| *Role physical* | 133 | 7 |
| *Bodily pain* | 135 | 3 |
| *General health* | 129 | 2 |
| *Vitality* | 135 | 3 |
| *Social functioning* | 136 | 2 |
| *Role emotional* | 134 | 5 |
| *Mental health* | 135 | 5 |
| *Physical component score* | 129 | 5 |
| *Mental component score* | 130 | 7 |
| **Self-related health according to the visual analogue scale in the EQ-5D questionnaire** | 145 | 11 |
